# Sodium Bicarbonate Toxicity: An Unusual Yet Potential Cause of Severe Metabolic Alkalosis

**DOI:** 10.7759/cureus.76949

**Published:** 2025-01-05

**Authors:** Mário Gil Fontoura, Luís Fernandes, Filipe Machado, Penelope Almeida, Teresa Pereira

**Affiliations:** 1 Internal Medicine, Unidade Local de Saúde de Entre Douro e Vouga, Santa Maria da Feira, PRT; 2 Internal Medicine, Hospital Lusíadas Porto, Porto, PRT; 3 Internal Medicine, Hospital da Lapa, Porto, PRT; 4 Intermediate Care Unit, Unidade Local de Saúde de Entre Douro e Vouga, Santa Maria da Feira, PRT

**Keywords:** acid-base disorders, electrolyte imbalances, metabolic alkalosis, over-the-counter antacids, sodium bicarbonate toxicity

## Abstract

Sodium bicarbonate, commonly known as baking soda, is present in various over-the-counter medications sold to the general public, including for the treatment of gastrointestinal symptoms such as nausea and dyspepsia. Less well known to the public are the severe complications associated with these treatments and their overuse, which can lead to significant toxicity, including metabolic alkalosis and associated electrolyte imbalances, further perpetuating or even worsening the acid-base disorder.

In this case report, we present a 45-year-old woman with a history of locally invasive breast cancer, who underwent primary chemotherapy and radiotherapy with remission. She presented to the Emergency Department with nausea, vomiting, and abdominal discomfort that began one month earlier, along with asthenia and weight loss predating these symptoms. She had begun taking over-the-counter medications recommended by a local herbalist, some of which contained sodium bicarbonate or calcium carbonate. The patient was hypotensive, vomiting, and showed signs of dehydration. Her blood gas analysis revealed severe metabolic alkalosis with significant hypokalemia and hypochloremia. Prompt treatment was initiated, and the patient was admitted to the Intermediate Care Unit. Following an exhaustive diagnostic workup for her initial symptoms, pyloric stenosis caused by a locally advanced gastric neoformation was identified and later confirmed to be carcinoma. The patient was referred to the Oncology Group, where primary chemotherapy was recommended and initiated; however, the patient ultimately succumbed to her illness.

In conclusion, recognizing and understanding the mechanisms underlying the generation and maintenance of metabolic alkalosis, along with its causes and a thorough review of the patient's medical history, are crucial for clinicians to ensure proper and timely intervention and correction of the acid-base disorder.

## Introduction

Sodium bicarbonate (NaHCO_3_), commonly known to the public as baking soda, is widely included in various manufacturers’ antacids, which are extensively used by the general population to treat nausea, dyspepsia, and heartburn [1]. Antacid misuse is the most common cause of bicarbonate overuse [1]. Sodium bicarbonate is widely available and easily accessible to the general population, as many antacids on the market do not require a prescription. Being a strong base, it acts as a buffer for hydrochloric acid (HCl), reducing gastric acidity and making it effective for patients with heartburn and dyspepsia. Although rare and not widely recognized, the overuse of sodium bicarbonate carries the risk of severe and potentially fatal toxicity. It can cause metabolic alkalosis and significant disturbances in acid-base and electrolyte balance, such as hypernatremia, hypokalemia, hypochloremia, and hypocalcemia [2-4]. Furthermore, its use can obscure underlying pathologies, as demonstrated in the following case report.

## Case presentation

A 45-year-old female presented to the Emergency Department with a five-week history of nausea, vomiting, asthenia, and approximately 5 kilograms of weight loss, accompanied by the onset of dyspnea in the last week. She denied diarrhea or other gastrointestinal symptoms as well as cardiac, other respiratory, neurologic, or genitourinary symptoms.

Medical history

The patient had a past medical history of locally invasive left breast cancer (cT3N3aM0 - TNM staging) [5], diagnosed in 2011 at 34 years of age. She was treated with primary chemotherapy and radiotherapy, achieving remission in 2012. She was subsequently discharged from an Oncology follow-up in 2020. No other relevant medical history was reported. She reported that since her cancer diagnosis, she experienced occasional nausea without vomiting. However, in the last month, with the onset of more severe symptoms, including *de novo* vomiting and asthenia, she began self-medicating with over-the-counter drugs, initially not disclosing this information to the attending clinician. She kept using these drugs as needed until this hospital admission.

Physical examination and diagnostic exams

On physical examination, the patient had a body mass index (BMI) of 19 kg/m², blood pressure of 89/48 mmHg, heart rate of 105 bpm, peripheral oxygen saturation (SpO₂) of 97% on room air, respiratory rate of 14 breaths per minute, and body temperature of 36.5 °C. Her skin and mucous membranes were dehydrated and pale, with no peripheral edema, findings most consistent with clinical hypovolemia.

The admission arterial blood gas (ABG) revealed severe metabolic alkalemia (pH: 7.81) and respiratory acidosis (pCO₂: 56 mmHg) with significant alkalosis (HCO₃⁻: 87 mmol/L). Electrolyte abnormalities included severe hypokalemia (K⁺: 2.1 mmol/L) and hypochloremia (Cl⁻: 62 mmol/L). Serum laboratory findings showed microcytic hypochromic anemia (hemoglobin: 10.4 g/dL), acute kidney injury (AKI) based on the Acute Kidney Injury Network (AKIN) criteria [6] (stage 2) with a serum creatinine of 3.9 mg/dL and urea of 98 mg/dL, ionized calcium of 0.86 mmol/L, and bilirubin of 1.36 mg/dL. No significant elevation in inflammatory markers was noted. Urinalysis demonstrated a urine pH of 8.00, which was consistent with the degree of alkalemia, mild proteinuria, and no other significant abnormalities. These findings were suggestive of hypovolemia, in conjunction with the noted acid-base disorder. An electrocardiogram (ECG) was performed, which showed no significant abnormalities. Obstructive renal and post-renal causes of acute kidney injury were excluded through renal ultrasonography, which showed no other notable findings. This suggested that a pre-renal cause of AKI was most likely.

A further investigation into the patient's medication use revealed that she had been taking several over-the-counter medications purchased from a local herbalist, two of which contained sodium bicarbonate and magnesium carbonate, both commonly used for nausea and vomiting.

Treatment, admission, and evolution

Given the patient’s severe metabolic alkalosis, resulting from both recurrent vomiting and the intake of bicarbonate-containing supplements, along with concomitant hypovolemia and electrolyte imbalances, intravenous fluid replacement was initiated. At the Emergency Department, the treatment included 2000 mL daily of intravenous sodium chloride (NaCl) 0.9% and potassium supplementation with 60 mEq of potassium chloride (KCl) administered at 125 mL/h, in addition to symptomatic treatment for nausea with intravenous metoclopramide (10 mg) and ondansetron (8 mg). This intervention led to a favorable response, with the patient demonstrating improved normotension and significant resolution of metabolic alkalosis and hypokalemia upon repeat laboratory evaluation. Although vomiting persisted to a degree, there was a notable reduction in its severity.

The patient was subsequently admitted to the Intermediate Care Unit (ICU) for further evaluation and continued management. Spot urine analysis showed the following electrolyte concentrations: sodium (Na⁺) 109 mEq/L, potassium (K⁺) 48.2 mEq/L, and chloride (Cl⁻) <20 mEq/L. The fractional excretion of sodium (FENa) was 2.1%, which was most consistent with an intrinsic renal injury. However, considering her prior fluid resuscitation with NaCl, likely chronic progression of renal dysfunction, and findings suggestive of hypovolemia, a pre-renal cause leading to intrinsic kidney injury was considered the most probable etiology. These findings also supported a diagnosis of chloride-responsive metabolic alkalosis, guiding careful fluid and electrolyte management, which resulted in stabilization of the patient’s hemodynamics and correction of both acid-base and electrolyte abnormalities over the course of hospitalization (Table 1).

**Table 1:** Acid-base parameters and electrolytes during hospital stay Legend: Ca2+, calcium; Cl-, chloride; HCO3-, bicarbonate; K+, potassium;  Na+, sodium; PaCO2, partial pressure of carbon dioxide; PaO2, partial pressure of oxygen; std, standardized

Laboratory parameters	Emergency department	Day 1 ICU	Day 5 ICU	Reference range
Ions				
Na^+ ^(mmol/L)	134	141	135	136-145 mmol/L
K^+ ^(mmol/L)	2.1	3.2	3.5	3.5-5.1 mmol/L
Cl^- ^(mmol/L)	62	-	101	98-107 mmol/L
Ca^2+^ ionized (mmol/L)	0.91	1.21	1.09	1.15-1.27 mmol/L
Renal Function				
Creatinine (mg/dL)	3.9	2.9	0.8	0.6-1.1 mg/dL
Urea (mg/dL)	98	90	28	15-40 mg/dL
Arterial blood gas				
pH	7.81	7.66	7.43	7.35-7.45
PaO2 (mmHg)	77	59	96	83-108 mmHg
PaCO2 (mmHg)	56	73	34	35-48 mmHg
HCO3^-^(std) (mmol/L)	87	59.6	22.6	21-28 mmol/L

**Figure 1:**
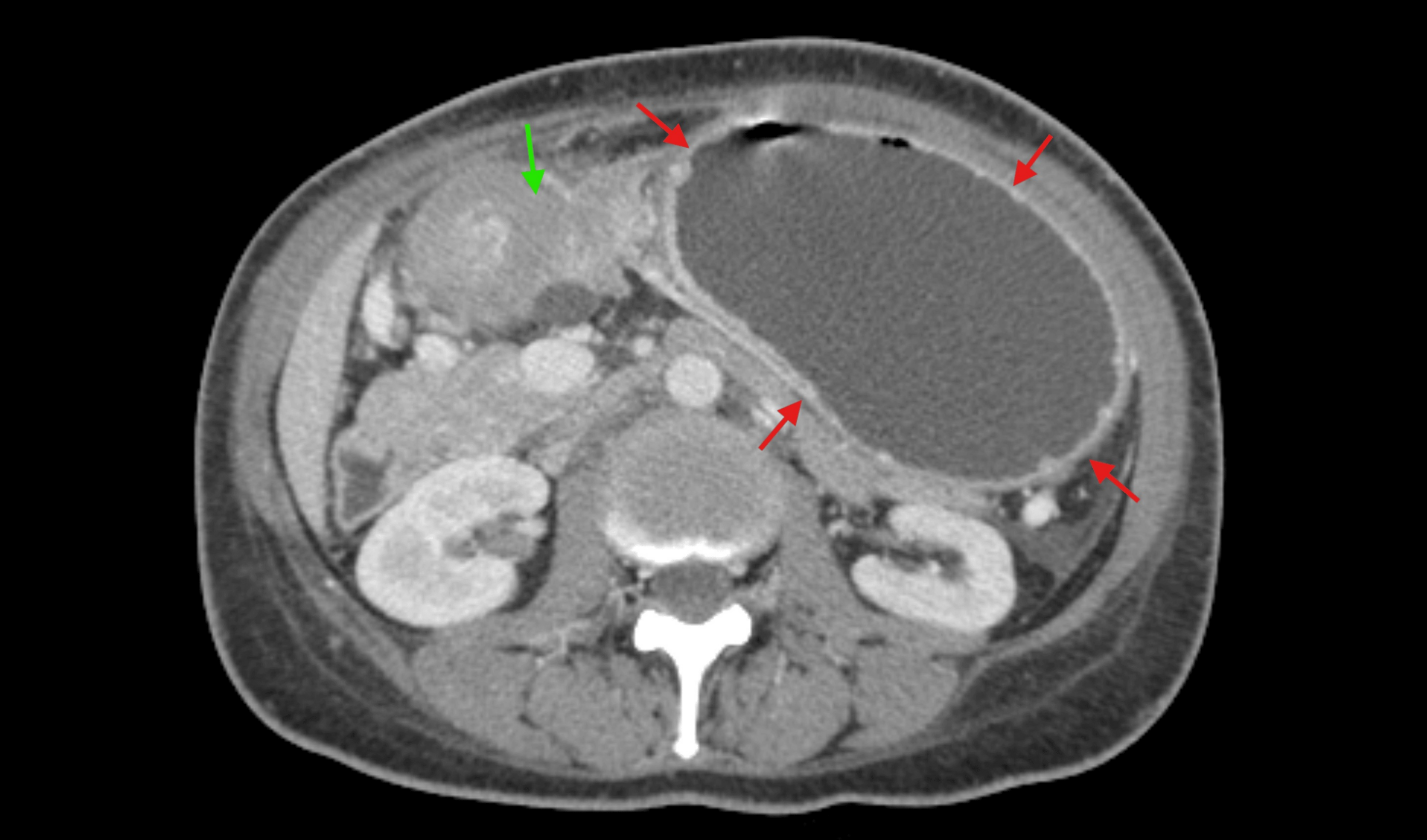
Axial CT scan of the abdomen revealing parietal thickening extending from the lesser curvature of the gastric antrum to the pylorus (green arrow) and significant gastric distension (red arrows)

**Figure 2:**
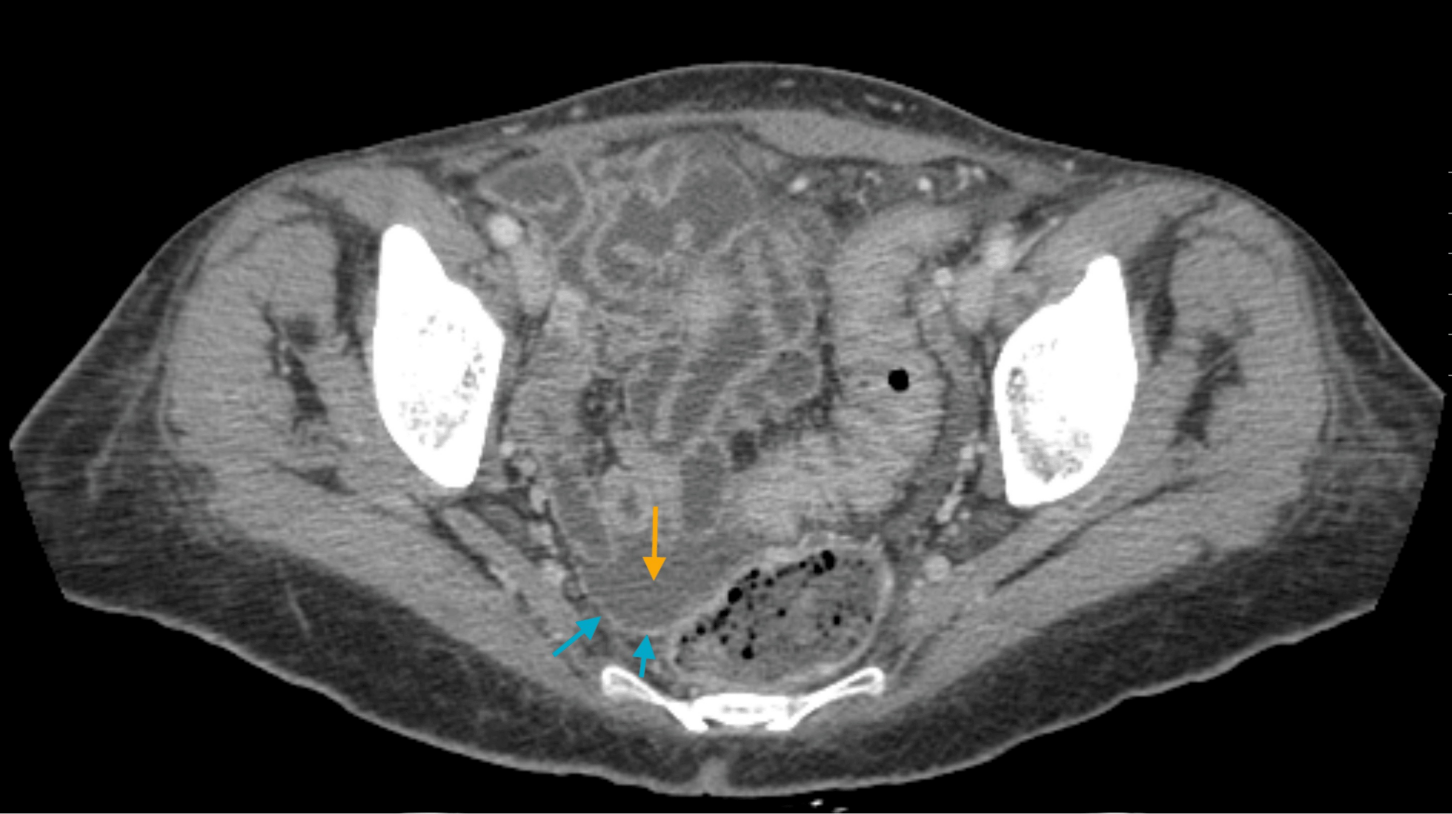
Axial CT scan of the pelvis showing irregular peritoneal thickening (blue arrows) and a small amount of peritoneal fluid (yellow arrow), findings suggestive of peritoneal carcinomatosis

Given the patient’s ongoing gastrointestinal symptoms, including asthenia, weight loss, and vomiting of uncertain origin, and considering her history of breast cancer, an extensive workup for a potential gastrointestinal neoplasm was undertaken. Tumor markers CEA and CA 19-9 were negative. An upper gastrointestinal endoscopy revealed pyloric stenosis. To further assess the stenosis and evaluate differential diagnoses, a contrast-enhanced computed tomography (CT) scan of the abdomen and pelvis was performed, which revealed significant parietal thickening extending from the small curvature of the antrum to the pylorus, as well as abnormal fat stranding near the celiac trunk and minimal peritoneal fluid (Figure 1 and Figure 2). These findings prompted an urgent exploratory laparoscopy, which revealed diffuse peritoneal carcinomatosis and an advanced gastric neoplasm. A biopsy of the mass was performed, and specimens were sent for pathological examination.

Outcome and follow-up

Subsequent pathological examination confirmed the presence of structures strongly suggestive of carcinoma, characterized by a diffuse architectural pattern and signet ring cells. The patient was then transferred to the Surgery ward for further management and was provided with appropriate guidance before being discharged after 34 days of hospitalization. She was referred to the Oncology Group for consultation and was recommended to undergo primary chemotherapy; however, she ultimately passed away.

## Discussion

Initially, the patient presented with severe metabolic alkalosis, which could not be solely attributed to vomiting. The delayed identification of the cause was due to the patient withholding information regarding self-administered medications. Upon disclosure of all medications taken, including sodium bicarbonate, a few symptoms were noted that preceded the use of some of these medications, raising the possibility of a paraneoplastic syndrome. This consideration ultimately guided the diagnostic workup presented earlier.

Serum and urine electrolyte levels are crucial in determining whether metabolic alkalosis is chloride-sensitive or resistant and whether it will respond to fluid resuscitation [7]. This information was instrumental in guiding the patient's volume management, which contributed to achieving hemodynamic stability and euvolemia. Additionally, the electrolyte imbalances associated with metabolic alkalosis were promptly addressed to prevent potential multiorgan complications.

From a pathophysiological perspective, several mechanisms contributed to the development and maintenance of metabolic alkalosis in this case. These included hydrogen ion loss due to vomiting, an intracellular shift of hydrogen ions secondary to severe hypokalemia, and the exogenous administration of sodium bicarbonate found in the patient’s over-the-counter medications [8,9]. Furthermore, impaired bicarbonate excretion was expected due to reduced arterial blood volume, a common consequence of persistent vomiting, which frequently leads to hypovolemia and metabolic alkalosis. Concurrent hypokalemia also contributed to increased renal ammoniagenesis and ammonium excretion, further impairing bicarbonate excretion.

## Conclusions

Over-the-counter medications, such as those used for the treatment of various symptoms, including dyspepsia, are generally deemed safe and are available without a medical prescription, often outside of pharmacies. An example of this, as highlighted in this case report, is baking soda. This accessibility facilitates their use but also increases the potential for misuse, as sodium bicarbonate can lead to severe acid-base disturbances and electrolyte imbalances, which may necessitate hospitalization and medical intervention. Additionally, self-treatment with over-the-counter drugs may delay seeking appropriate medical care and, as demonstrated in this case, may mask underlying medical conditions.

Therefore, healthcare providers must conduct a thorough medical history and carefully confirm all medications being used to avoid severe complications. It is equally important that patients are educated on the correct dosage (posology) of these medications and the associated risks.
